# Safety and Efficacy of Stereotactic Body Radiation Therapy for Locoregional Recurrences After Prior Chemoradiation for Advanced Esophageal Carcinoma

**DOI:** 10.3389/fonc.2020.01311

**Published:** 2020-07-31

**Authors:** Steven N. Seyedin, Margaret K. Gannon, Kristin A. Plichta, Laith Abushahin, Daniel J. Berg, Evgeny V. Arshava, Kalpaj R. Parekh, John C. Keech, Joseph M. Caster, James W. Welsh, Bryan G. Allen

**Affiliations:** ^1^Department of Radiation Oncology, University of Iowa Hospitals and Clinics, Iowa, IA, United States; ^2^Carver College of Medicine, University of Iowa, Iowa, IA, United States; ^3^Division of Hematology/Oncology, Department of Internal Medicine, University of Iowa Hospitals and Clinics, Iowa, IA, United States; ^4^Division of Thoracic and Cardiovascular Surgery, Department of Surgery, University of Iowa Hospitals and Clinics, Iowa, IA, United States; ^5^Department of Radiation Oncology, The University of Texas MD Anderson Cancer Center, Houston, TX, United States

**Keywords:** esophageal cancer, recurrence, SBRT, inoperable, chemoradiation

## Abstract

**Purpose:** This study aimed to investigate the feasibility of stereotactic body radiation therapy (SBRT) as salvage therapy for locally recurrent esophageal cancer. We hypothesized that SBRT would provide durable treated tumor control with minimal associated toxicity in patients with progressive disease after definitive radiation, chemotherapy, and surgical resection.

**Methods:** This single-institution retrospective study assessed outcomes in patients who received SBRT for locoregional failure of esophageal cancer after initial curative-intent treatment. Only patients who had received neoadjuvant chemoradiation (≥41.4 Gy) for esophageal cancer were selected. Subsequent surgical resection was optional but institutional follow-up by an oncologist was required. The primary endpoints of this study were gastrointestinal and constitutional toxicity, scored with the Common Terminology Criteria for Adverse Events v5.0. A secondary outcome, treated-tumor control, was assessed with RECIST v1.1.

**Results:** Nine patients (11 locoregional recurrences) treated with SBRT were reviewed, with a median follow-up time of 10.5 months. Most patients initially presented with T3 (88.9%), N1 (55.6%), moderately differentiated (66.7%) adenocarcinoma (88.9%), and had received a median 50.4 Gy delivered over 28 fractions with concurrent carboplatin/paclitaxel chemotherapy followed by surgical resection. Median time to recurrence was 16.3 months. Median total dose delivered by SBRT was 27.5 Gy (delivered in five fractions). Two patients experienced acute grade 1 fatigue and vomiting. No patient experienced grade 3 or higher toxicity. One patient experienced failure in the SBRT treatment field at 5.8 months after treatment and six patients developed distant failure. The median progression-free survival time for SBRT-treated tumors was 5.0 months, and median overall survival time was 12.9 months.

**Conclusions:** This single-institution study demonstrated the feasibility of SBRT for locoregional recurrence of esophageal cancer with minimal treatment-related toxicity and high rates of treated tumor control. Prospective studies identifying ideal salvage SBRT candidates for locoregional failure as well as validating its safety are needed.

## Introduction

Approximately 17,650 new cases of esophageal cancer are diagnosed annually in the United States, and about 50% of those cases present as locally advanced disease (stages IIb–IIIc) (1). For patients who can tolerate it, the standard of care is neoadjuvant radiation and chemotherapy (CRT) followed by surgical resection, otherwise known as trimodality therapy (TMT) (2). Despite the aggressiveness of TMT, 15–20% of patients will experience locoregional recurrence within 36 months of surgery (3–7). Rates of locoregional failure increase to 50% for those with inoperable disease, with most failures appearing within the irradiated field (8). Local recurrence of esophageal cancer may present with significant pain, bleeding, vomiting, obstruction, or dysphagia (2).

Palliative treatment strategies for locally recurrent or progressive disease after CRT or TMT include multiagent chemotherapy, repeat CRT, radiation alone, and salvage esophagectomy (9–11). However, many patients will be ineligible for aggressive salvage therapy owing to poor performance status, development of concomitant distant failure, and history of prior esophagectomy (10, 12). Therefore, palliative options that provide durable local control with minimal treatment-associated toxicity are needed.

Stereotactic body radiation therapy (SBRT) allows the precise delivery of high-dose radiation, usually in 3–5 fractions, to small target areas (13, 14). Several studies have shown promising results from using SBRT to treat residual, recurrent, and oligometastatic lung and colorectal cancers, among others (15–18). The role of SBRT in the treatment of recurrent esophageal cancer is currently unknown. We retrospectively assessed the safety and efficacy of SBRT for recurrent or progressive esophageal cancer.

## Methods and Materials

### Patients

This IRB-approved, retrospective single-institution study (#201701826) evaluated outcomes for patients who had received SBRT for one or more locoregional failures after initial TMT for esophageal cancer from July 2016 through January 2018. All patients must have received at least 41.4 Gy of radiation during initial CRT. Esophageal recurrence was confirmed by biopsy or chest computed tomography (CT). Extent of recurrent disease after CRT was assessed by positron emission tomography (PET) or CT of the chest, abdomen, and pelvis. Eligibility for salvage SBRT was determined by an institutional thoracic multidisciplinary tumor board and included having locally limited disease as well as being ineligible for surgical salvage because of prior esophagectomy or poor performance status.

### Stereotactic Body Radiation Therapy

**Figure 1:**
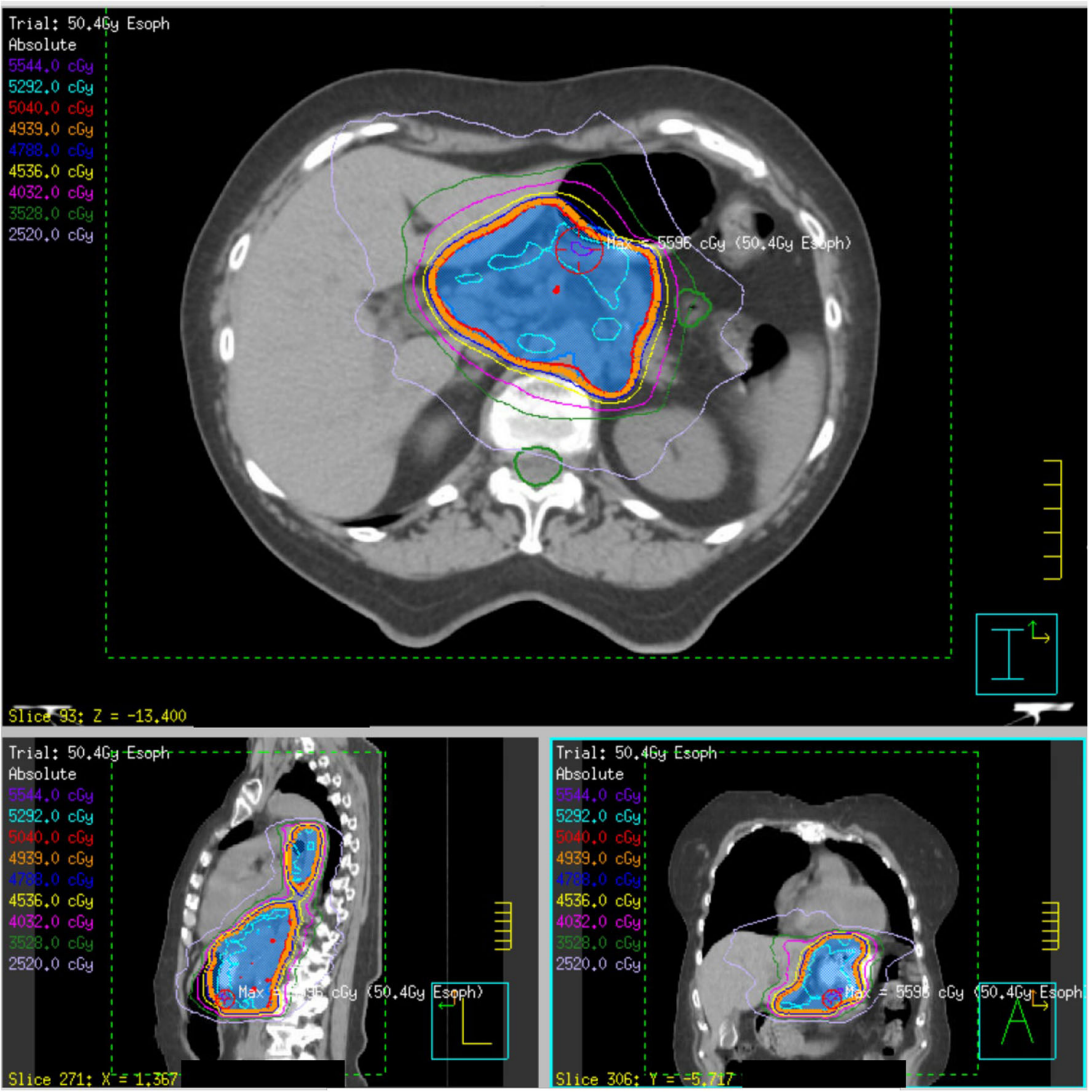
Initial definitive radiation plan for a T3N1 lower esophageal adenocarcinoma (patient 4) of 50.4 Gy delivered over 28 fractions. The PTV50.4 volume, indicated in blue colorwash, is encompassed within the red 50.4 Gy isodose line.

**Figure 2:**
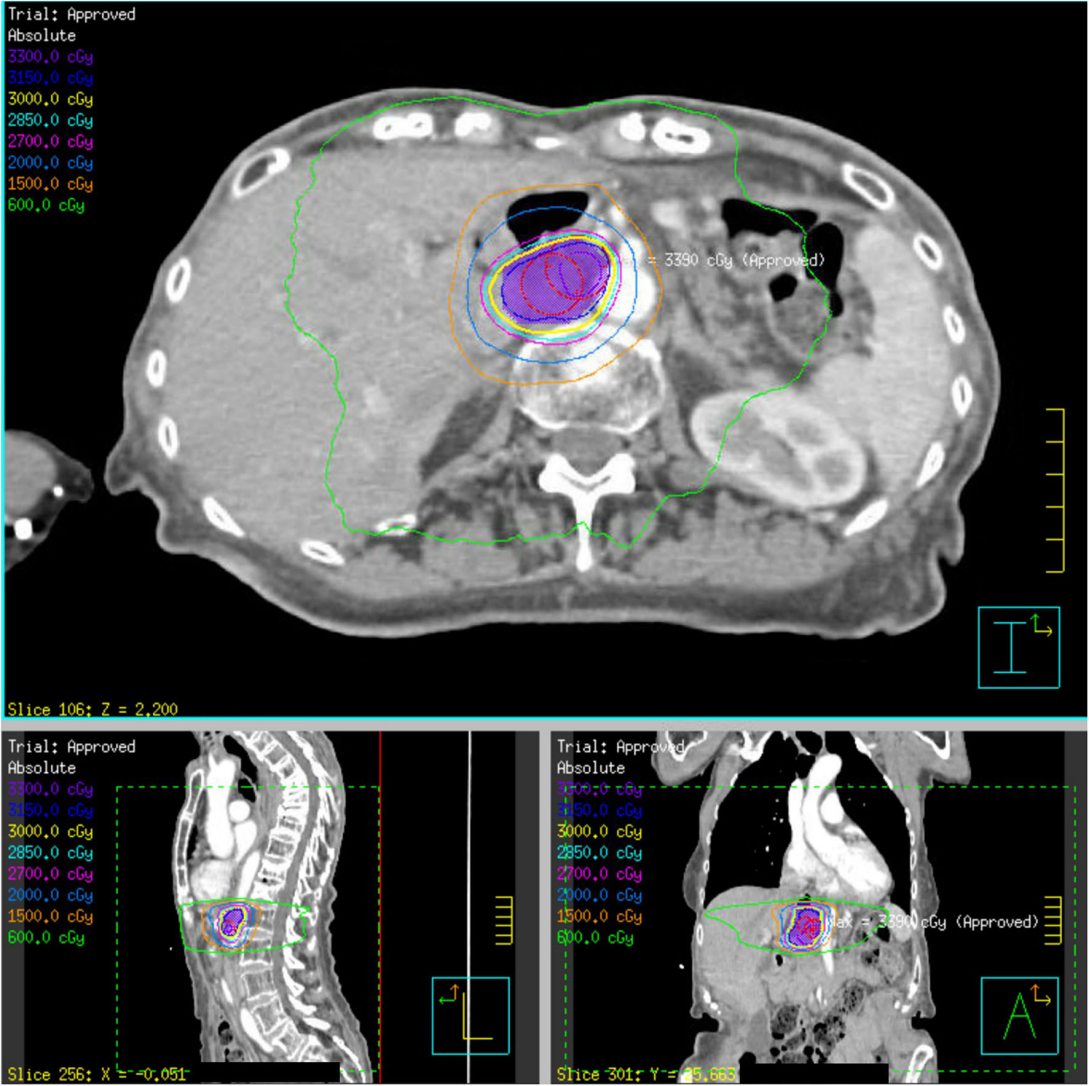
SBRT plan of 30 Gy delivered over five fractions to a recurrent gastrohepatic node within the initial 50.4 Gy field (patient 4). The PTV30 volume, indicated in purple colorwash, is encompassed within the yellow 30 Gy isodose line.

**Figure 3:**
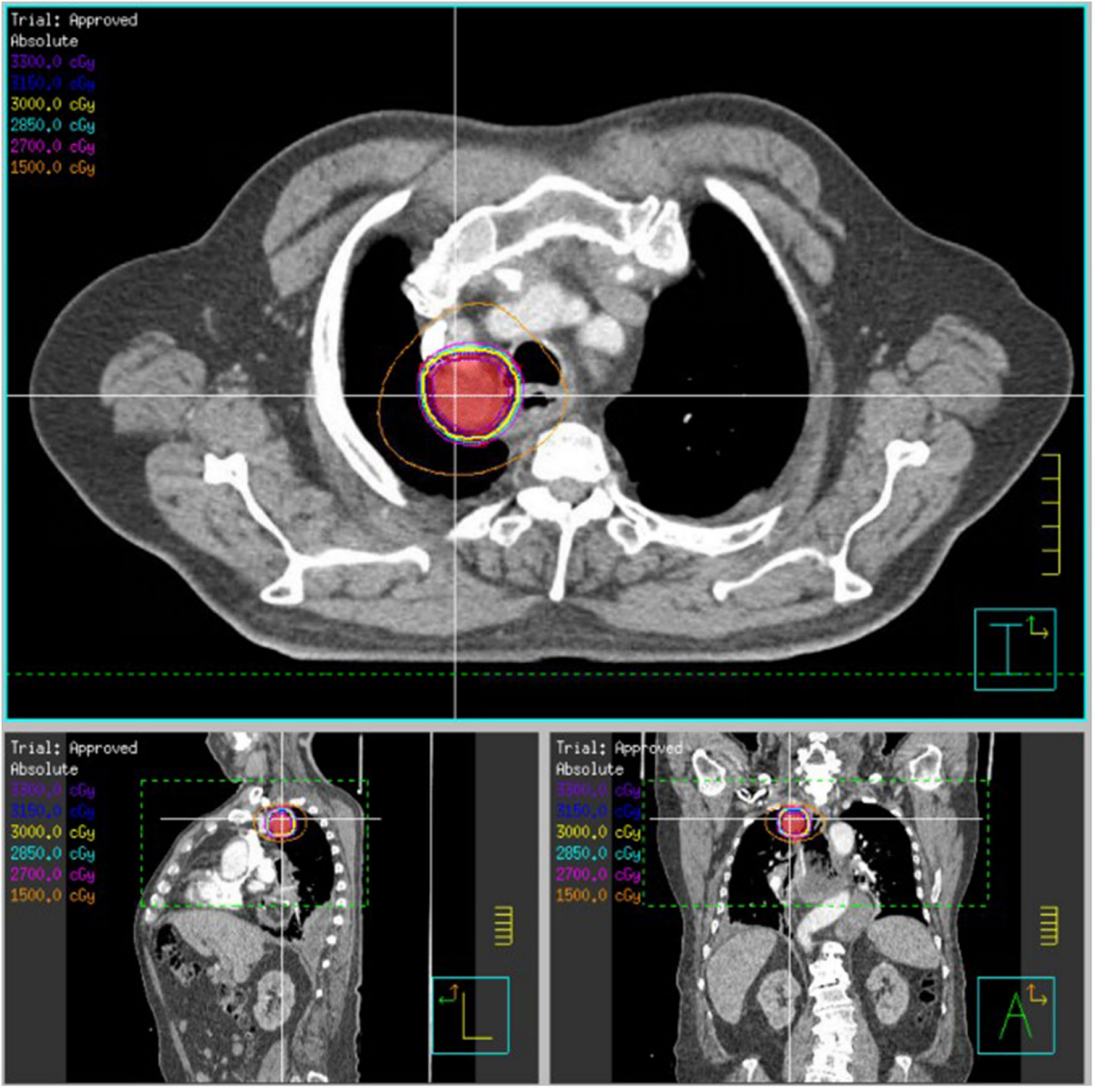
SBRT plan of 30 Gy delivered over five fractions to an esophageal/mediastinal squamous cell carcinoma recurrence (patient 2). The patient previously received 50.4 Gy delivered over 28 fractions. The PTV30, indicated in red colorwash, is encompassed within the yellow 30 Gy isodose line.

**Figure 4:**
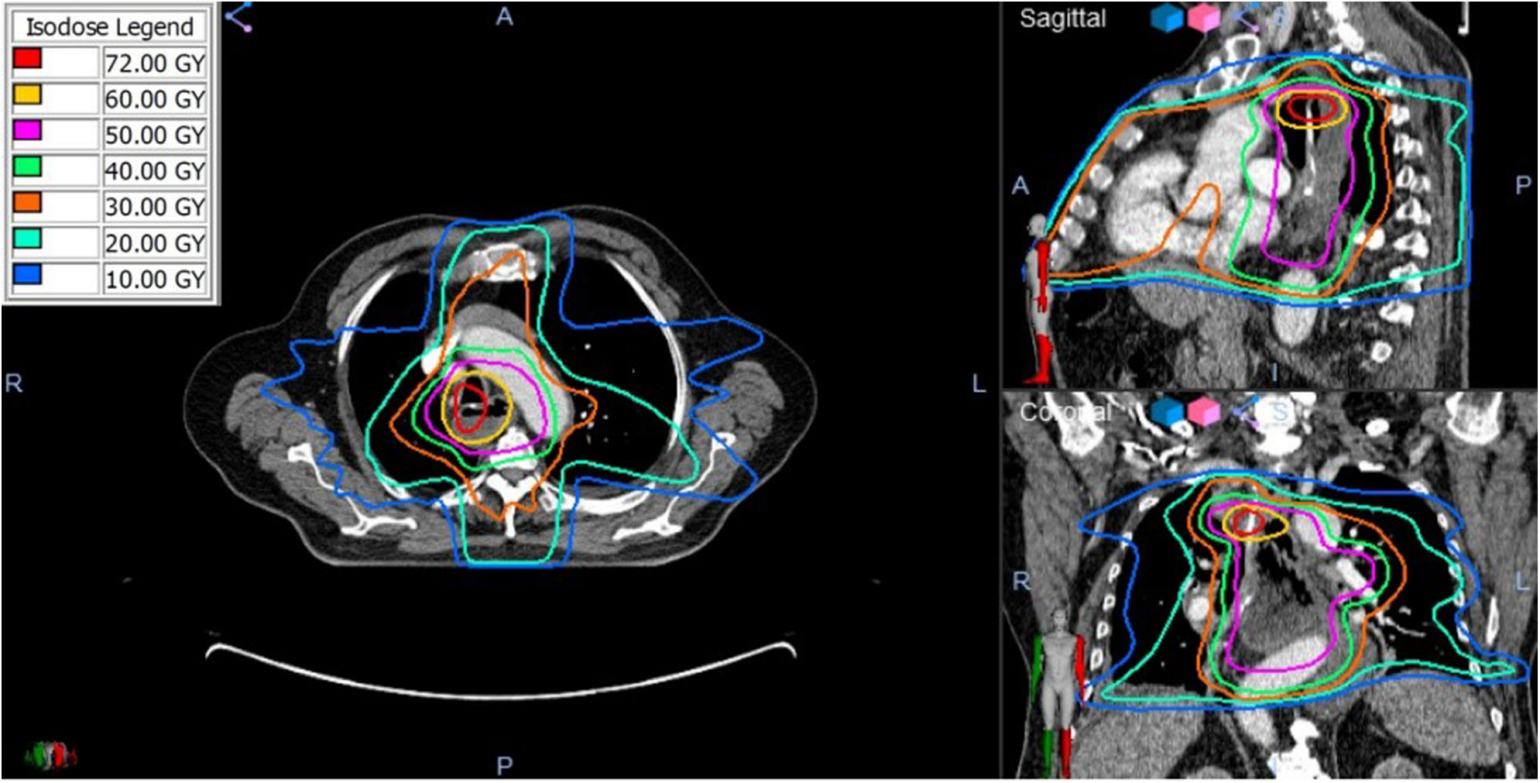
The composite radiation dose plan using Velocity (Varian) Deformable Registration to combine the radiation doses from the initial and retreatment SBRT plans. Significant dose overlap is present demonstrated by the total cumulative doses >50 Gy.

Initial simulation for all salvage SBRT plans included 4D CT scanning to account for tumor motion during the various breathing phases, and all patients underwent PET to further delineate the primary gross tumor volume. The 4D CT data sets included 10 respiratory phases ranging from 20 to 100% inspiration to 0–80% expiration. Gross tumor volumes from the 100% inspiration, 0% expiration, and full-expiration breath hold datasets were combined to create the internal target volume (ITV). Gating was implemented when the extent of tumor motion was more than 1 cm. The planning target volume (PTV) consisted of the ITV plus a 5-mm margin. Table 1 provides the treatment volumes (GTV, ITV, and PTV), conformality index, and dose inhomogeneities generated using this treatment planning approach. SBRT was delivered with a 7-MV flattening filter-free beam in five fractions given every other day, which is typical at [the treating institution]. The minimum SBRT dose required was 22.5 Gy; the median dose (given in five fractions) was 27.5 Gy (range 22.5–30 Gy). The radiation dose from the initial CRT plans was considered when evaluating dose constraints for salvage SBRT, as was the previous dose given specifically to the area of recurrence to be treated with SBRT. For patients who received the initial CRT at centers other than that where the salvage SBRT was given, PDFs of dosimetric plans were reviewed to approximate the dose previously given to the area of recurrence and to the nearby organs at risk (Figures 1, 2); for patients given both CRT and SBRT at the same institution, previous radiation doses were calculated with Velocity software (Varian Medical Systems, Atlanta GA; Figures 3, 4). Dose constraints applied were as follows: spinal cord, V28 < 0.03 cm^3^, V22 < 0.35 cm^3^, and V15.6 < 1.2 cm^3^; trachea and ipsilateral bronchus, V40 < 0.03 cm^3^, V32 < 5 cm^3^; heart, V38 < 0.3 cm^3^, and V32 < 15 cm^3^); and gastric pull-up area, V35 < 0.05 cm^3^, V26.5 < 5 cm^3^. Time to recurrence after SBRT was calculated from the SBRT completion date.

**Table 1:** Treatment volumes, conformality index, and dose inhomogeneity.

**Patient code**	**GTV volume (cc)**	**ITV volume (cc)**	**PTV volume (cc)**	**Conformality index^c^**	**Dose inhomogeneity^d^**
1^a^	9.67	16.85	51.97	0.99	1.25
	4.28	6.43	16.20	1.14	1.2
2	10.58	16.14	32.52	1.01	1.18
3	6.96	8.57	19.13	1.12	1.21
4	3.59	6.76	26.70	1.15	1.13
5	11.40	15.81	46.70	1.12	1.24
6	7.83	8.84	21.88	1.08	1.21
7	4.41	N/A^b^	11.88	1.09	1.23
8	19.68	N/A^b^	55.27	0.98	1.18
9^a^	46.93	51.57	117.92	1.19	1.25
	10.99	12.05	34.83	1.2	1.15

a *Two relapse sites treated with SBRT*.

b *Tumor motion assessed at the time of simulation was minimal so 4D-CT imaging was not used to create an ITV*.

c *Conformality index is the ratio of the prescription isodose volume to the PTV volume*.

d *Dose inhomogenity is the ratio of the maximum dose within the tumor to the prescribed dose*.

### Toxicity and Treatment Response Evaluation

The primary aim of this study was to determine the safety of SBRT for recurrent esophageal cancer. Information on gastrointestinal and constitutional toxicity during and after SBRT was extracted from the medical records and scored with the Common Terminology Criteria for Adverse Events v5.0. Evaluated toxic effects included fatigue, nausea, vomiting, small-intestine ulcers or perforations, gastroparesis, gastrointestinal/iliac obstruction, and gastric ulcers. Toxicity assessments recorded at [the treating institution] during follow-up by either a radiation, surgical, or medical oncologist were included in this study. Toxic effects occurring within 3 months of the start of SBRT were defined as acute, and those occurring >3 months afterward were classified as chronic. ^18^F-fluorodeoxyglucose-PET or CT scans were obtained every 3–6 months after SBRT (or sooner if indicated by symptoms) and those scans were used to assess treatment response. A secondary endpoint was treated tumor control after SBRT, which was determined by RECIST v1.1 (19).

### Statistical Analysis

Progression-free survival (PFS) and overall survival (OS) were calculated from the start of SBRT until the date of progression and death or last visit with an oncologist, and analyzed with IBM SPSS version 25 (Chicago, IL).

## Results

### Patients

Nine patients (with 11 locoregional recurrences) treated with SBRT were identified for analysis. The median follow-up time was 10.5 months, and the median patient age was 63 years. Most patients had initially presented with T3 (8), N1 (5), moderately differentiated (6) adenocarcinoma (8), and had received 50.4 Gy in 28 fractions with concurrent carboplatin/paclitaxel chemotherapy (Table 2). One patient (patient 7) did not undergo surgical resection after neoadjuvant CRT upon the discovery of positive retroperitoneal lymph nodes at that time as well as the development of bilateral pulmonary embolism requiring anticoagulation 1 month later. As for the other eight patients, two had McKeown resection, five had transhiatal esophagectomy, and one had an esophagectomy of unknown type at an outside institution. Pathologic evaluation after esophagectomy revealed negative margins in six patients, <1 mm margins in two patients, and residual positive lymph nodes in five patients.

**Table 2:** Patient and tumor characteristics.

**Patient ID**	**Age, years**	**Sex**	**Tumor histology**	**Tumor grade**	**Tumor location**	**Disease stage**	**Primary therapy**
1^a^	64	M	SCCa	MD	Lower	T3N0M0	TMT
2	72	M	SCCa	PD	Lower	T3N1M0	TMT
3	59	M	AC	PD	Lower	T2N0M0	TMT
4	68	F	AC	MD	Lower	T3N1M0	TMT
5	51	M	AC	MD	Lower	T3N1M0	TMT
6	50	M	AC	WD	Lower	T3N3M0	TMT
7	64	M	AC	MD	Middle	T3N2M1	CRT only
8	59	M	AC	MD	GE junction	T3N1M0	TMT
9^a^	61	M	AC	MD	Lower	T3N1M0	TMT

*SCCa, squamous cell carcinoma; MD, moderately differentiated; PD, poorly differentiated; WD, well differentiated; TMT, trimodality therapy; CRT, chemoradiation*;

* *Same patient treated for two sites of locoregional recurrence*.

a *Two relapse sites treated with SBRT*.

For the eight patients who received TMT, recurrence developed at a median interval of 16.3 months (range 3.2–58.6 months) after esophagectomy; recurrence in the other patient appeared at 10.4 months. Sites of recurrence included mediastinal lymph nodes (*n* = 7), gastrohepatic lymph node (*n* = 1), esophagectomy anastomotic site (*n* = 1), celiac axis (*n* = 1), and left supraclavicular node (*n* = 1; Table 3). Four recurrences (in patients 1, 3, 5, and 8) were outside the original CRT field. Only two patients were given systemic therapy at the time of recurrence (patient 1 was given anti-PD1 [pembrolizumab] and patient 8 was given FOLFOX). Patients underwent SBRT at a median 17.3 months after esophagectomy (*n* = 8) or prior CRT (*n* = 1).

**Table 3:** Failures after primary esophageal cancer treatment.

**Patient ID**	**Time to failure, months**	**Confirmed by**	**Tumor location**	**Estimated initial dose overlap to the area, Gy**	**SBRT Dose, Gy (delivered in 5 fractions)**	**Systemic therapy after SBRT (in months)**
1	16	CT scan	Celiac axis, mediastinum (*n* = 2)	41.4^a^	30^a^	Pembrolizumab (3.7)
2	58.6	Biopsy	Esophageal/mediastinum	42	30	None
3	35.3	Biopsy	Paratracheal node	None	27.5	None
4	14.1	Biopsy	Gastrohepatic node	50.4	30	None
5	43.4	Biopsy	Anastamotic site	None	27.5	Pembrolizumab (17.7)
6	16.6	Biopsy	Paratracheal node	50.4	27.5	Pembrolizumab (4.0)
7	10.4	Biopsy	4L lymph node	5	30	FOLFOX (2.4)
8	3.2	Biopsy	Left supraclavicular lymph node	None	25	FOLFOX (3.1)
9	14.7	Biopsy	Mediastinum (*n* = 2 lesions)	45^b^	22.5^b^	None

a *Dose overlap was 41.4 Gy for the recurrence in the celiac axis but 0 Gy for the mediastinal recurrence; SBRT dose to both lesions was 30 Gy*.

b *Dose overlap was 45 Gy for both lesions in the mediastinum; SBRT dose to both lesions was 22.5 Gy*.

### Toxicity

All patients were able to complete SBRT with minimal side effects during treatment; two patients experienced acute grade 1 fatigue and one patient experienced acute grade 1 vomiting (Table 4). One patient experienced chronic grade 1 nausea at 4.5 months after SBRT. None of the patients on this study experienced grade 3 or higher toxicity, and no patients developed acute or chronic ulcerations or perforations, gastroparesis, or gastrointestinal obstructions after SBRT.

**Table 4:** Toxicity after SBRT salvage therapy.

**Patient IS**	**Acute toxicity**	**CTCAE grade**	**Chronic toxicity**	**CTCAE grade**	**CTCAE Total**
1	None	–	None	–	0
2	Vomiting	1	Nausea	1	2
3	None	–	None	–	0
4	Fatigue	1	None	–	1
5	None	–	None	–	0
6	None	–	None	–	0
7	None	–	None	–	0
8	None	–	None	–	0
9	Fatigue	1	None	–	1

*CTCAE, Common Terminology Criteria for Adverse Events*.

### Treated Tumor Control and Survival

**Figure 5:**
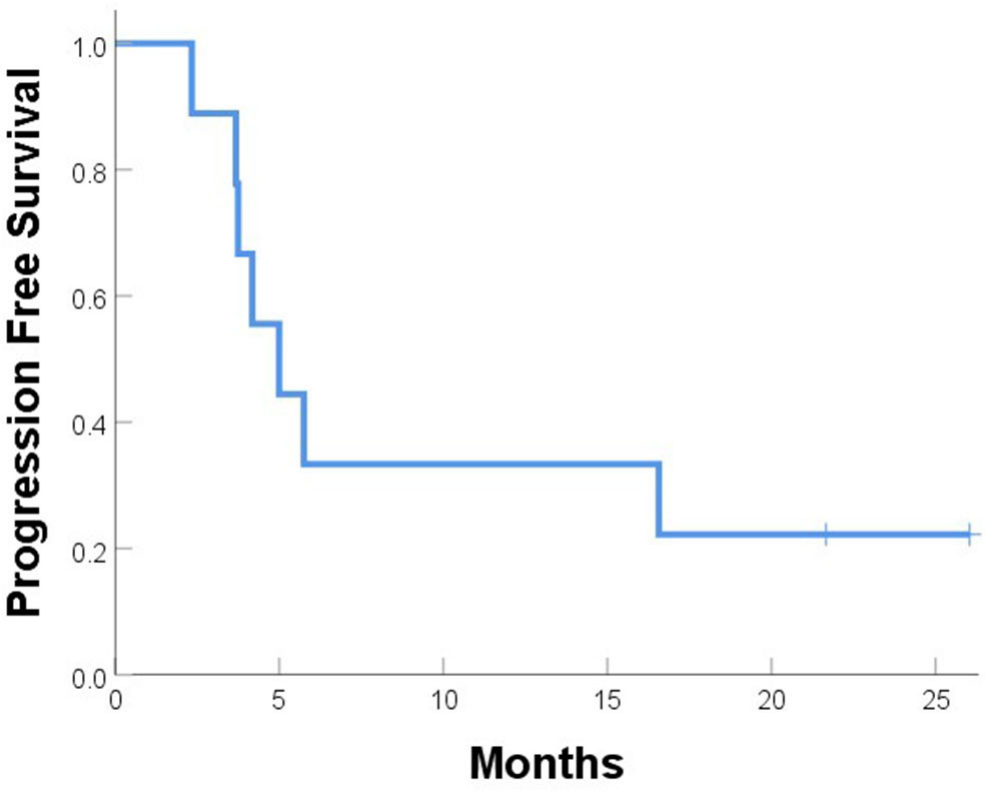
Progression-free survival after SBRT for recurrent or progressive esophageal cancer.

**Figure 6:**
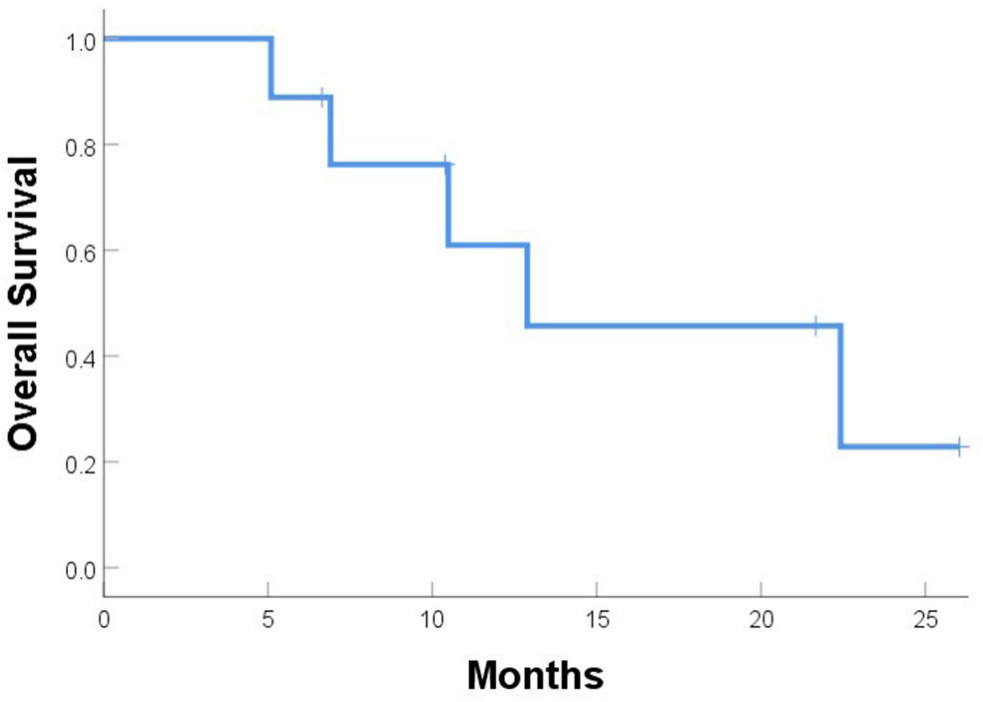
Overall survival after SBRT for recurrent or progressive esophageal cancer.

One patient (patient 9) experienced simultaneous SBRT-treated tumor failure and distant failures in the liver and pancreas at 5.8 months after completing SBRT. Six patients developed distant failure at various sites: at 2.3, 3.7, 3.7, 4.2, 4.9, and 16.6 months after SBRT including the liver, 4L nodes, periaortic nodes, retroperitoneal nodes, supraclavicular nodes, bone, and adrenal glands. At a median follow-up of 10.5 months, the median PFS time after SBRT was 5.0 months, and the median OS time was 12.9 months; at the time of this analysis, five patients had died (Figures 5, 6). Five patients received treatment for progression after salvage therapy (3 pembrolizumab as part of a clinical trial and 2 FOLFOX), and four patients did not receive any additional treatment. The mean time between the end of SBRT and initiation of systemic therapy for relapse was 6.2 months (range 2.4–17.7). Two patients were alive with no evidence of disease.

## Discussion

To our knowledge, this is the largest retrospective study to date of patients who received SBRT for locoregional recurrence after CRT for locally advanced esophageal cancer (20–22). We observed that salvage SBRT yielded high rates of treated tumor control and survival rates comparable to those after other salvage treatment options, with minimal toxicity (33% G1 toxicity and 0% G3+ toxicity) (21, 23–25). Indeed, SBRT was well-tolerated, with only two patients developing acute grade 1 fatigue, one experiencing acute grade 1 vomiting, and one experiencing chronic grade 1 nausea. No gastrointestinal ulcerations, perforations, strictures were observed.

Considering that the highest dose of SBRT given was 30 Gy in five fractions, these low rates of toxicity were not surprising and are consistent with other retrospective studies noting minimal toxicity from SBRT for recurrences after previous definitive CRT in head and neck or lung cancer (15, 26). Most of the published results on SBRT-related esophageal toxicity come from the non-small cell lung cancer literature. One retrospective study of 52 patients with PTVs within 2 cm of the esophagus noted that significant grade ≥3 toxicity occurred when point doses exceeded 51 Gy and 1 cm^3^ doses exceeded 48 Gy (27). In a similar study at Memorial Sloan Kettering Cancer Center, 125 patients experienced esophageal toxicity after SBRT when primary or metastatic lung tumors were within 2 cm of the proximal bronchial tree or when the PTV intersected medial structures (28); however, only two patients experienced grade 3 events related to SBRT.

We found in this small study that SBRT could provide effective control of treated recurrent tumors, as only 1 of the 11 treated tumors recurred within the SBRT field (in patient 9). Eight of the nine patients in this study had received TMT as primary therapy. Sudo et al. (29) examined 27 patients who experienced local failure after TMT. Of those 27 patients, 4 underwent salvage surgery, all of whom experienced considerable morbidity and two required additional surgery for failure of the anastomosis; another 11 patients received chemotherapy (specific agents not listed), resulting in a median OS time of 5 months. That same group also evaluated the role of salvage therapy for locoregional failure after definitive CRT alone (10); in that study, for the 36% of patients who could undergo salvage surgery, the median OS time was 58.6 months from relapse. However, rates of surgical complications were again high, including pulmonary events (17%), anastomotic leak (17%), and readmission to intensive care (17%), and two patients (9%) died within 90 days of surgery. Although salvage surgery can lead to OS of 30–45% at 5 years (30), the patients in our study were not considered eligible for repeat surgery by our multidisclipinary thoracic tumor board. Another advantage to SBRT, in addition to avoiding perioperative risks, is that SBRT can be combined with checkpoint inhibitors, which can prolong systemic disease-free survival. A recent retrospective study of two trials investigating either anti-PD1 or anti-CTLA4 agents given with SBRT for metastatic non-small cell lung cancer found that SBRT increased the efficacy of PD1 therapy by 98% relative to historical controls (31). Presumably this approach could have benefitted the patients in this study, as 5 of 6 developed distant failure within 5 months after SBRT.

Several groups have also investigated the use of multiagent chemotherapy for recurrent esophageal disease (11, 25, 32). Although response rates to these regimens range from 40 to 60%, they also confer high rates of hematologic and gastrointestinal toxicity. Several groups have also investigated the role of CRT as salvage therapy. In one phase II study testing platinum and fluorouracil with conventionally fractionated radiotherapy to 60 Gy for 30 patients with locoregional recurrence after resection (33), the 5-year local control rate was 71.5% and the median OS time was 21 months. However, patients in that study had not received neoadjuvant CRT, which likely would have increased the efficacy of salvage CRT. Another group retrospectively evaluated the effectiveness of repeat CRT after CRT alone for 39 patients with locoregional recurrence; in that study weekly paclitaxel/carboplatin was given with radiation to 50.4 Gy delivered over 28 fractions (23). At a median follow-up interval of 15 months, the locoregional failure rate at 1 year after salvage CRT was 21%; however, one patient died of grade 5 neutropenic infection and 39% experienced grade 3 toxicity requiring a temporary nasogastric feeding tube. In short, although repeat CRT can be effective for controlling recurrent disease after bimodality therapy, repeat CRT can also have significant toxicity.

Other groups have investigated the benefit of radiation alone for locoregional recurrence after definitive CRT. Zhou et al. retrospectively compared salvage radiation therapy (54 Gy in 30 fractions) with chemotherapy or supportive care for 114 patients who developed local recurrences after CRT alone (24). At a median follow-up time of 20 months, receipt of radiation was associated with a significant benefit in OS (21.8 vs. 8.3% at 3 years). However, ~20% of patients developed a fistula or perforation, which imparted a mortality rate of 16.4%. Another study examining outcomes for 30 patients given multifraction high-dose-rate brachytherapy found a median local PFS interval of 9.8 months and an OS rate of 31.5% at 1 year—but five patients experienced fatal toxicity (34). In contrast to these studies, our findings suggest that SBRT could provide similar rates of local control with low rates of toxicity.

A few case reports have also described SBRT for recurrent esophageal cancer after TMT. One group published their experience with a 58-year-old man who developed recurrence, manifested as dysphagia, at 4 years after TMT; that patient was given 35 Gy in seven fractions as salvage. By the final fraction, the dysphagia had significantly improved and the patient was able to resume normal intake; however, he died of metastatic disease at 11 months after SBRT (20). Another case report of two patients treated with 30 and 40 Gy in five fractions did not report any toxicity on follow-up, although the median follow-up time was not reported (21). These results coincide with our experience with SBRT and encourage further investigation of the use of SBRT for locally recurrent esophageal cancer.

Limitations of our study include its retrospective nature, short follow-up, and small number of patients, which could mask adverse events. Caution should be taken in interpreting this study's findings, given that eight of the nine patients underwent esophagectomy before SBRT, which would remove much of the previously irradiated tissue as well as sections of normal esophagus. Also, composite dosimetric plans combining initial CRT and SBRT plans to precisely quantify the maximum gastric pull up and esophageal radiation dose could be generated for two patients who had received initial CRT. In the seven other cases, PDFs of the initial CRT dosimetry were used to approximate previous dose to the area treated with SBRT. Therefore, this study cannot surmise how the dose of radiation given to the gastric pull-up impacted the safety of re-irradiation. Finally, while 8 of 11 recurrences treated were within the previously irradiated field, only two in the current study presented with a prior dose overlap of 50.4 Gy.

This study observed that one patient developed simultaneous local and distant recurrence, six developed distant recurrence, and two remained disease free post-SBRT treatment. Five of the patients who developed distant failure received chemotherapy after SBRT. Thus, a median OS of 12.9 months cannot be solely attributed to SBRT and emphasizes the importance of appropriate patient selection for salvage SBRT and/or chemotherapy. These data suggest that systemic therapy should be administered shortly after SBRT in patients with multisite recurrences. Trials demonstrating a survival benefit with consolidative local therapy in oligometastatic disease support this approach as most failures after treatment were distant (18, 35). Methods of detecting micrometastatic progression, such as circulating tumor DNA and tumor cells, may select patients who would benefit from immediate adjuvant chemotherapy after SBRT (36, 37).

In conclusion, this study showed encouraging initial results regarding the feasibility and safety of SBRT for recurrent inoperable esophageal cancer. While we only observed low-grade toxicities, this therapy is still investigational and should be offered as part of prospective safety-focused clinical trials. These results require validation with dosimetric data describing overlapping treatment fields (including dose to the gastric pull-up) before SBRT can be accepted as a safe treatment option for esophageal cancer recurrence. Approaches to improve patient selection that would benefit from SBRT with systemic therapy are needed.

## Data Availability Statement

The raw data supporting the conclusions of this article will be made available by the authors, without undue reservation.

## Ethics Statement

The studies involving human participants were reviewed and approved by University of Iowa Institutional Review Board. The patients/participants provided their written informed consent to participate in this study.

## Author Contributions

Patient data was obtained by SS and MG. KP performed the statistical analysis. MG, SS, and BA wrote the original manuscript with editorial input and further suggested data acquisition from all other authors. All authors contributed to various sections of the manuscript.

## Conflict of Interest

The authors declare that the research was conducted in the absence of any commercial or financial relationships that could be construed as a potential conflict of interest.
